# Supplementary material for: Explainable machine learning for profiling the immunological synapse and functional characterization of therapeutic antibodies
Source: Nat Commun. 2023 Nov 30;14:7888. doi: 10.1038/s41467-023-43429-2 (PMC10689847; doi:10.1038/s41467-023-43429-2)
Supplement: Supplementary file 1 — Supplementary Information [file 41467_2023_43429_MOESM1_ESM.pdf]

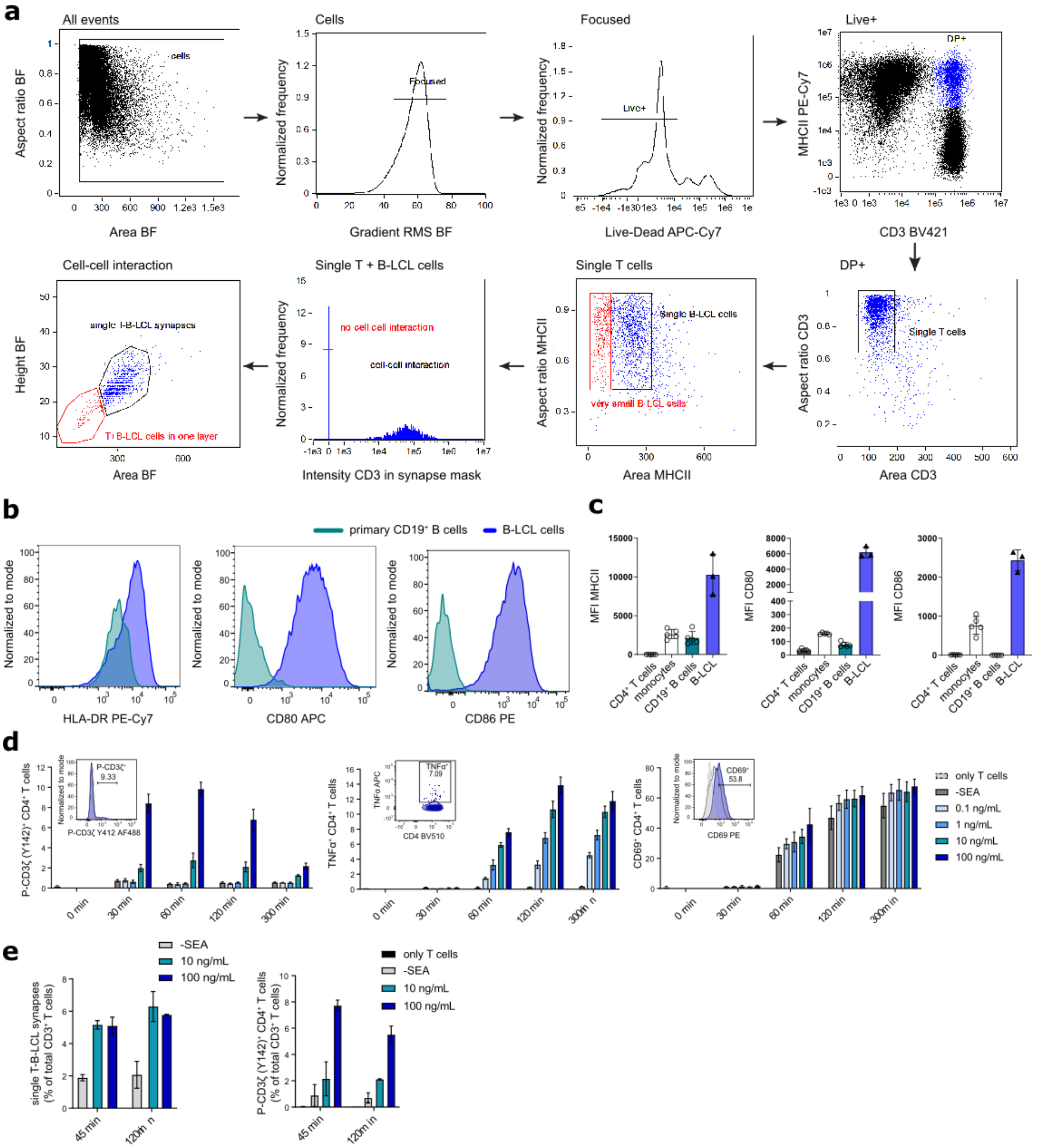

Supplementary Figure 1: Assay conditions to analyze immune responses between primary human CD4⁺ T cells and B-LCL cells using conventional and imaging flow cytometry.

a Gating strategy to identify single interacting T-B-LCL synapses using the IDEAS software of the imaging flow cytometer. A detailed description of the gating can be found in the Methods section.

b FACS histograms showing the surface-expression levels of MHCII (HLA-DR), CD80, and CD86 of primary CD19⁺ B cells from PBMCs and B-LCL cells. B-LCL cells express higher levels of the antigen-presenting receptor MHCII as well as of the co-stimulatory molecules CD80 and CD86 compared to primary B cells.

c Quantification (Mean fluorescence intensity, MFI) of MHCII, CD80 and CD86 surface expression on primary CD4$^+$ T cells, CD19$^+$ B cells, and monocytes from PBMCs and B-LCL cells. Data are from five donors in three independent experiments. The B-LCL cell line was analyzed in triplicates.

d Pre-testing of assay conditions using conventional FACS. Primary memory CD4$^+$ T cells isolated from PBMCs of healthy donors were stimulated with B-LCL cells in the presence of different concentrations of SEA (0.1-100 ng/mL) or left untreated (-SEA). Frequencies of P-CD3ζ$^+$, TNF-α$^+$ and CD69$^+$ CD4$^+$ T cells were determined at various time points. The small FACS histograms in the bar graphs show the expression levels of the three markers by comparing the highest concentration of SEA (100 ng/mL) with the untreated control (-SEA) after 60 min. The data shown represent one experiment using T cells from three different donors.

e Percentage of single T-B-LCL synapses and P-CD3ζ$^+$ CD4$^+$ T cells measured by imaging flow cytometry between two different SEA concentrations (10 and 100 ng/mL) after 45 and 120 min. Data represent two donors.

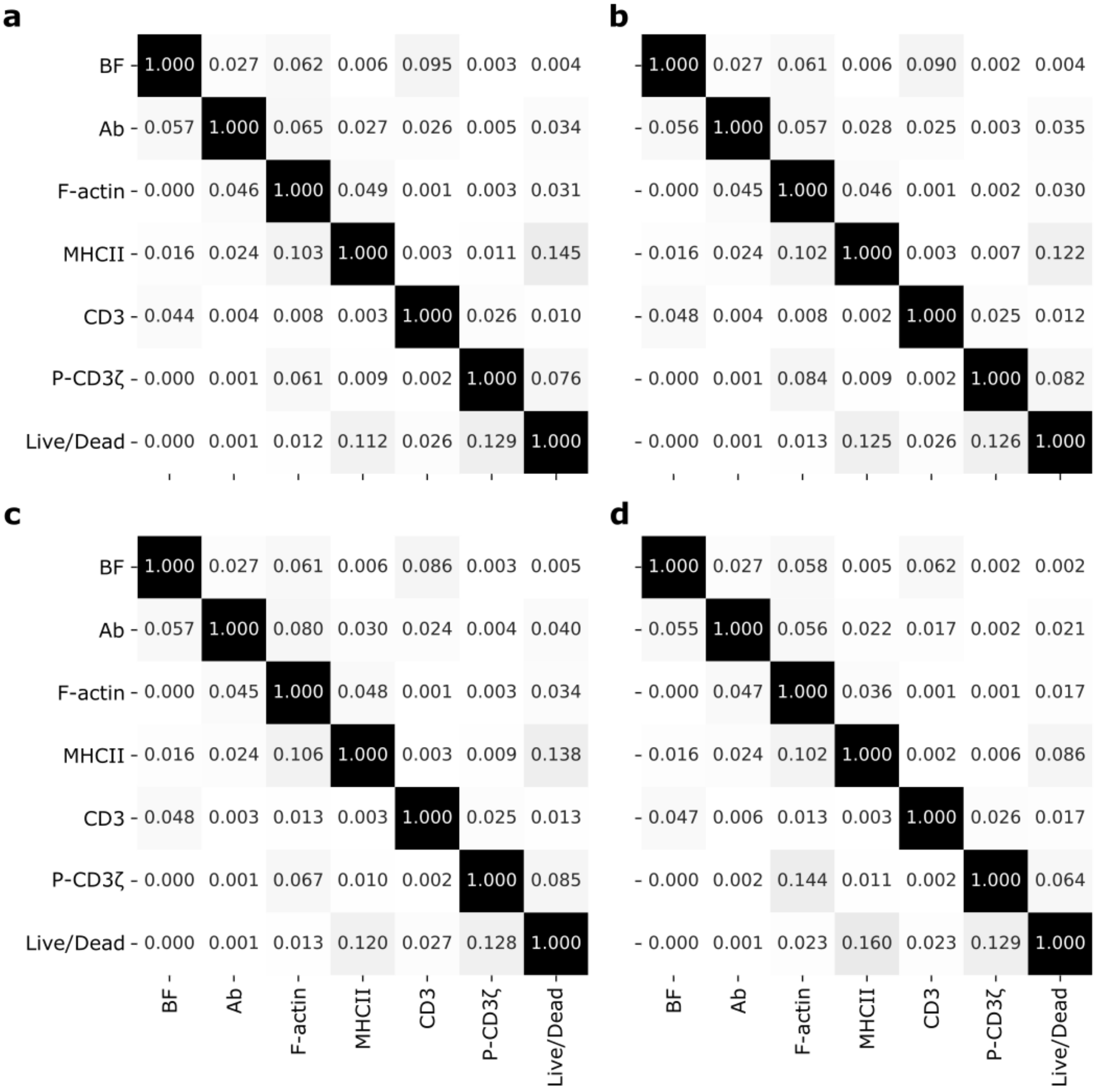

Supplementary Figure 2: Compensation matrices to minimize spillovers into the different channels for all experiments
For each experiment, a new compensation matrix was generated. Subfigures a-d represent experiments I-IV, respectively.

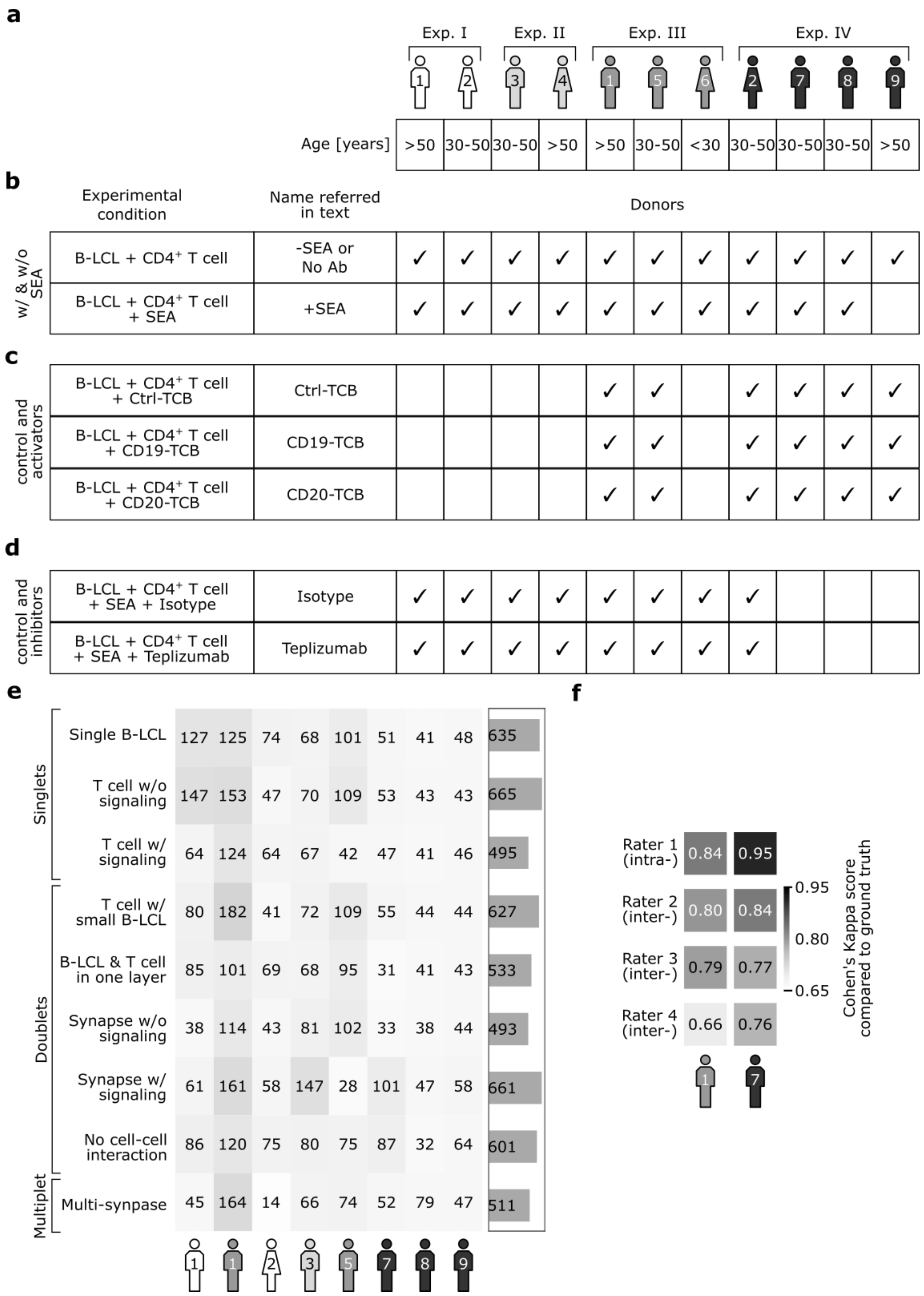
a
Exp. I
Exp. II
Exp. III
Exp. IV
Age [years]
>50 30-50 30-50 >50 >50 30-50 <30 30-50 30-50 30-50 >50
b
Experimental condition
Name referred in text
Donors
w/ & w/o SEA
B-LCL + CD4+ T cell
-SEA or No Ab
B-LCL + CD4+ T cell + SEA
+SEA
c
control and activators
B-LCL + CD4+ T cell + Ctrl-TCB
Ctrl-TCB
B-LCL + CD4+ T cell + CD19-TCB
CD19-TCB
B-LCL + CD4+ T cell + CD20-TCB
CD20-TCB
d
control and inhibitors
B-LCL + CD4+ T cell + SEA + Isotype
Isotype
B-LCL + CD4+ T cell + SEA + Teplizumab
Teplizumab
e
Singlets
Single B-LCL 127 125 74 68 101 51 41 48 635
T cell w/o signaling 147 153 47 70 109 53 43 43 665
T cell w/ signaling 64 124 64 67 42 47 41 46 495
Doublets
T cell w/ small B-LCL 80 182 41 72 109 55 44 44 627
B-LCL & T cell in one layer 85 101 69 68 95 31 41 43 533
Synapse w/o signaling 38 114 43 81 102 33 38 44 493
Synapse w/ signaling 61 161 58 147 28 101 47 58 661
No cell-cell interaction 86 120 75 80 75 87 32 64 601
Multiplet
Multi-synpase 45 164 14 66 74 52 79 47 511
f
Rater 1 (intra-) 0.84 0.95
Rater 2 (inter-) 0.80 0.84
Rater 3 (inter-) 0.79 0.77
Rater 4 (inter-) 0.66 0.76
0.95
0.80
0.65
Cohen's Kappa score compared to ground truth

mentary Figure 3. Donors and experiments information.

a List of the donors, their ages, gender, and experiment numbers used in this study.

b List of experiments and donors with and without SEA.

c List of experiments and donors with TCBs and their control.

d List of experiments and donors with Teplizumab and isotype control

e Number of labeled by expert data per donor.

f Intra- and inter-rater comparison of the annotation. Images from two donors were randomly selected and reannotated by four annotators to assess the quality of the original annotations (ground truth). Rater 1 and 2 are expert immunologists, and Rater 1 is the original annotator of the ground truth. There is a very strong agreement (Cohen kappa score>=0.80) between expert immunologists.

a

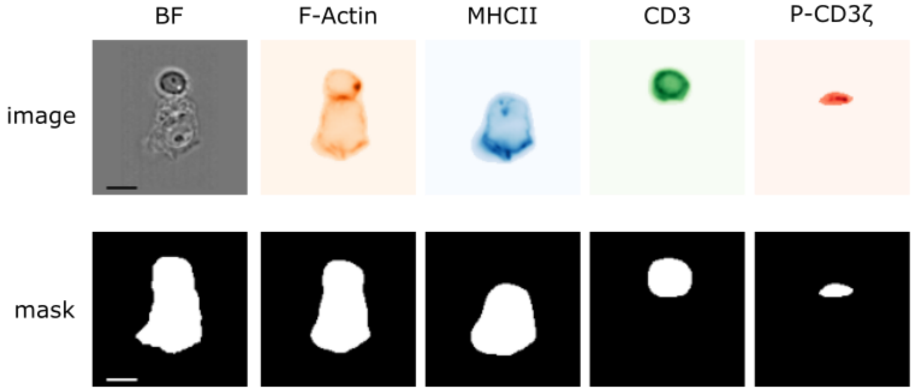

b

Morphology features
Input: image + mask

01. Cell area
02. Bounding box area
03. Convex area
04. Eccentricity
05. Equivalent diameter
06. Euler number
07. Extent
08. Feret diameter maximum
09. Filled area
10. Major axis length
11. Minor axis length
12. Hu moments
13. Orientation
14. Solidity
15. Weighted Hu moments

Synaptic features:
Input: image + mask

01. Enrichment of intensity in synaptic area (sum-based)
02. Enrichment of intensity in synaptic area (mean-based)
03. Enrichment of intensity in synaptic area (max-based)

Others:

01. Background mean
02. Gradient RMS

Intensity statistics:
Input: image + mask

01. Mean intensity
02. Standard deviation
03. Skewness
04. Kurtosis
05. Minimum intensity
06. Maximum intensity
07. Percentiles of intensity
08. Shannon entropy

Co-localization:
Input: two channels at a time

01. Correlation distance
02. Euclidean distance
03. Manders overlap coefficient
04. Structural similarity
05. Hausdorff distance

Texture features:
Input: image

01. Contrast
02. Dissimilarity
03. Homogeneity
04. ASM
05. Energy
06. Correlation

c

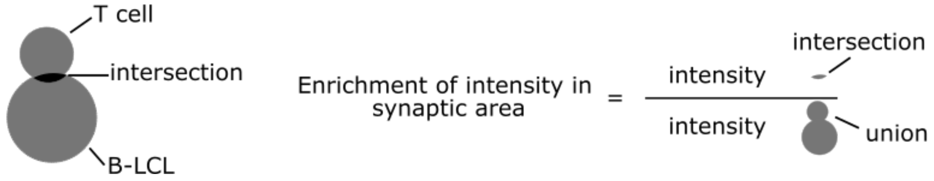

Supplementary Figure 4. scifAI interpretable feature list

a Visual representation of each multi-channel image and corresponding masks. Masks were exported along with images from the IDEAS software (scale bar = 2.4μm).

b List of all features implemented in scifAI. The morphology, intensity statistics, textures, and synaptic features are based on one channel. The colocalization features are based on two channels. scifAI automatically detects the existing channels and generates the specified features.

c Visual representation of how synaptic features are calculated. The ratio of intersection over the union of cells was used to calculate the enrichment of intensity in the synaptic area. The intersection is calculated using the masks from CD3 and MHCII channels. The sum of intensities in each region is calculated for the sum-based enrichment feature. The same logic applied to the mean-based and max-based enrichment features.

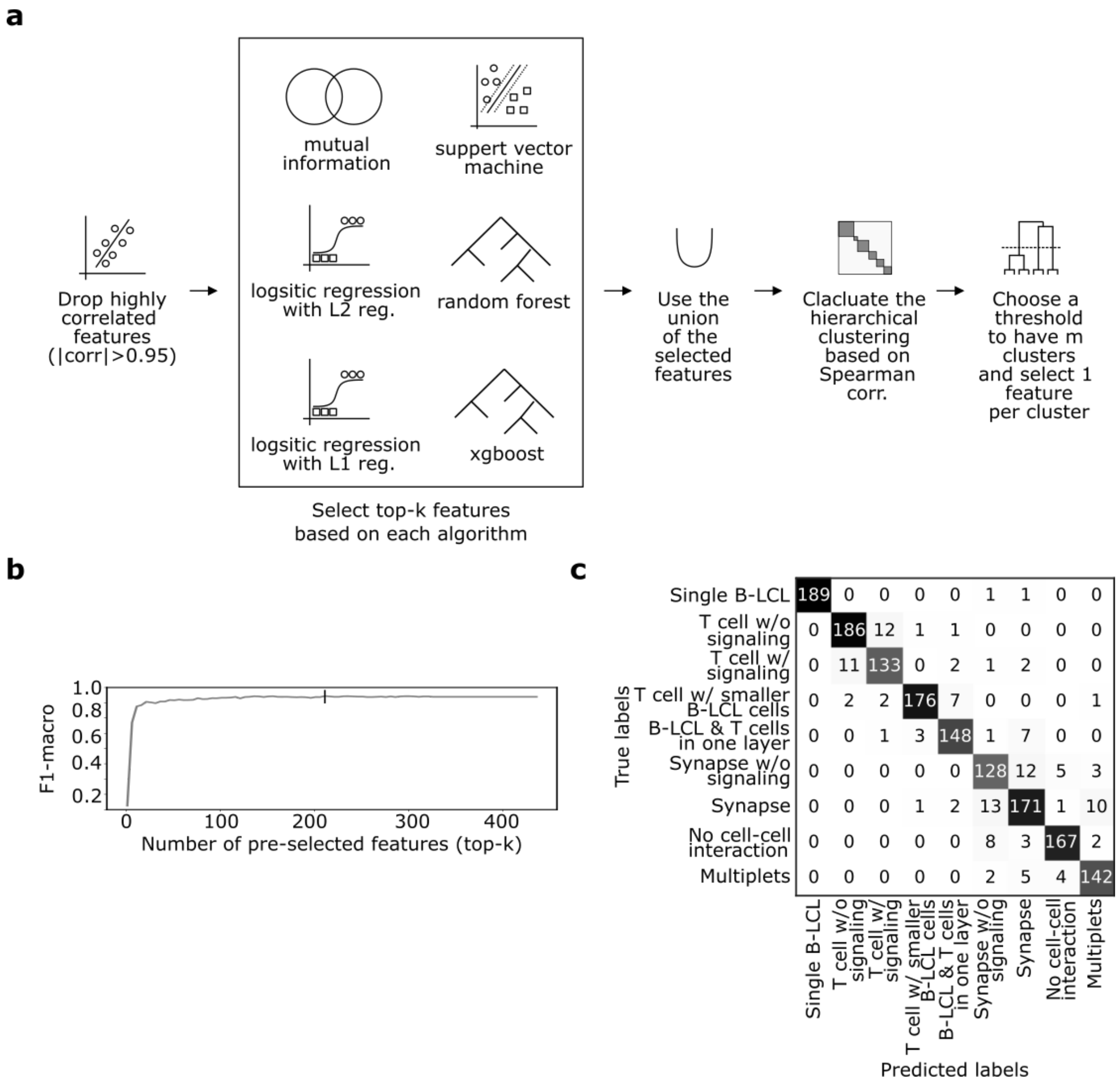

Supplementary Figure 5. Machine learning pipeline for classification

a Feature pre-selection pipeline to reduce the dimensionality of the feature space and remove multicollinearity. First, the highly correlated features are dropped. Then an ensemble of different classifiers is trained on the data, and their top-k features are selected. Finally, hierarchical clustering is done on top of the union of the features to account for multicollinearity.

b The optimal number of pre-selected features before passing the pre-selected features to the XGBoost classifier. To obtain the optimal top-k, the data selection pipeline + XGboost was trained on stratified randomly selected 85% of the training set and tested on the remaining 15%.

c Confusion matrix of the data selection pipeline (top-k = 211) + XGBoost, based on the predictions on the test set.

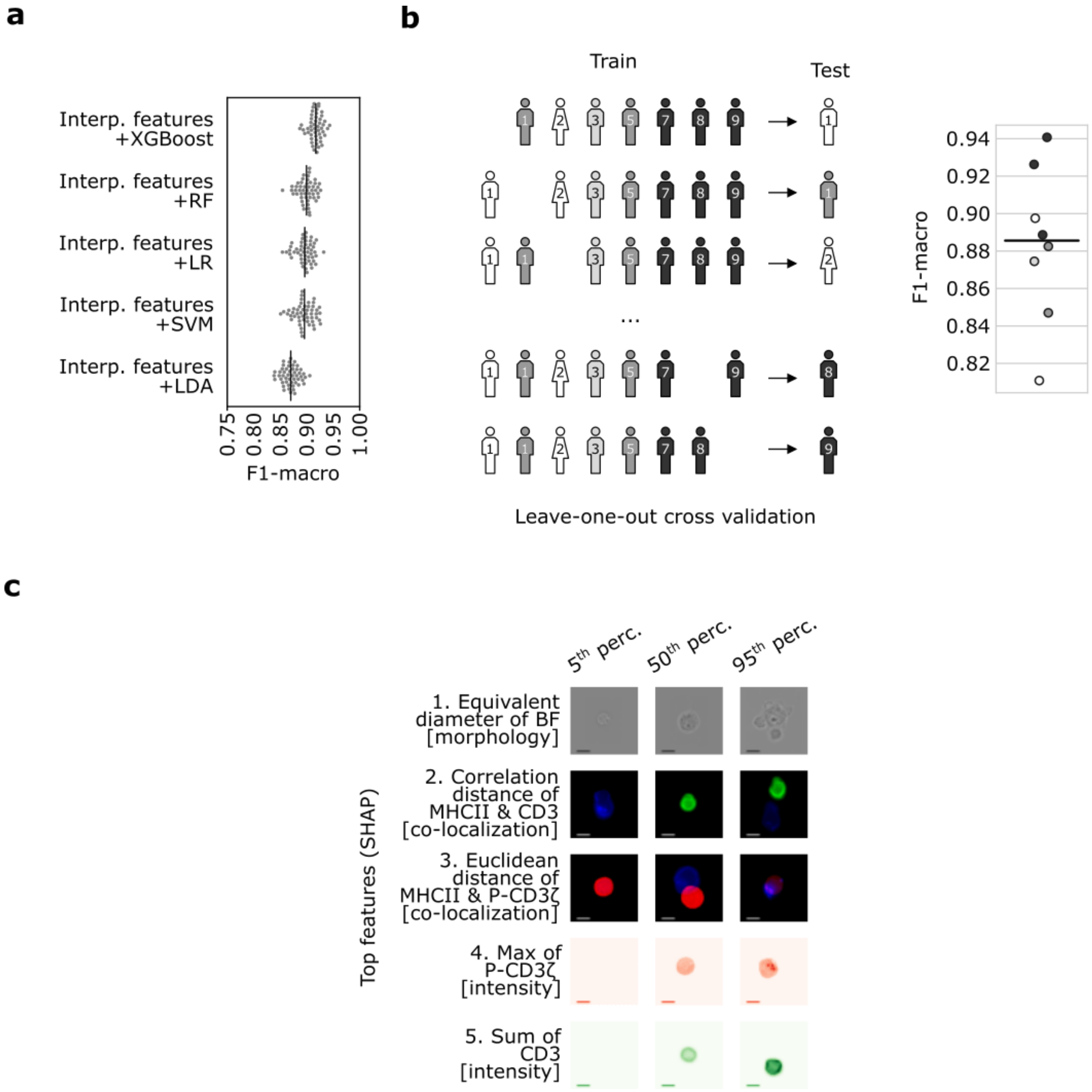

Supplementary Figure 6. Sanity check of the feature importance, interpretable features, and generalizability and efficiency of scifAI

a Ablation study: comparison of additional classifiers on the interpretable features, including XGboost, random forest (RF), logistic regression (LR), support vector machine (SVM), and linear discriminant analysis (LDA). While XGBoost exhibits the highest performance, other classifiers with different capacities show relatively similar performance, which suggests that interpretable features are the driving factor for good performance.

b Leave-one-donor-out cross-validation confirms that the combination of interpretable features and XGBoost can be generalized to new donors. Each point depicts the result on the test value.

c Top 5 features based on SHAP. The SHAP feature importance (post-model) method aligns with the previously ranked features based on the intrinsic feature importance from the XGboost model (in-model) shown in Fig. 1d (scale bar = 2.4μm).

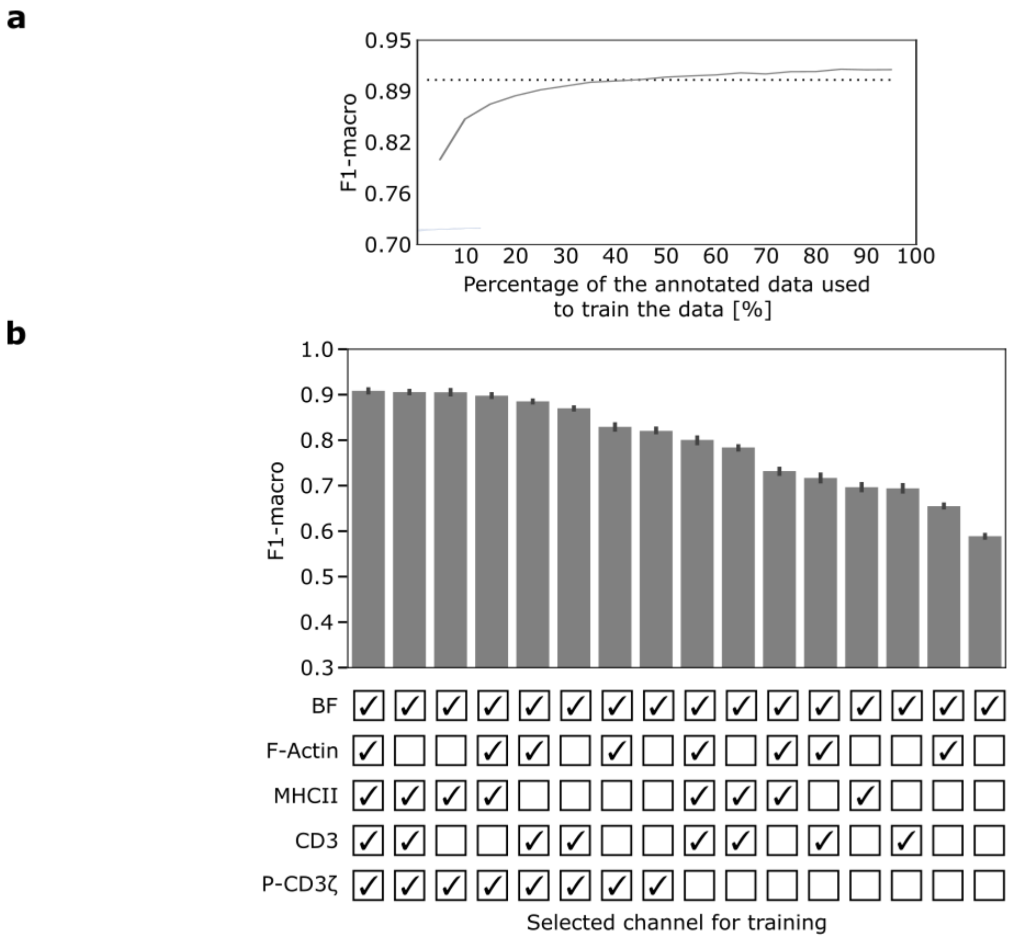

Supplementary Figure 7. Extracted information in the annotated data

a Number of annotated images vs. the classification performance

b The same XGBoost model was used to train the classifier. In each step, features based on the selected channels were used for training the classifier. As brightfield (BF) is a stain-free channel, it is always kept in the data. The combinations are ranked based on F1-macro. The combination of BF, MHCII, and P-CD3ζ (third from left) performs similarly to using all the channels. The barplots depict mean value with 95% confidence interval.

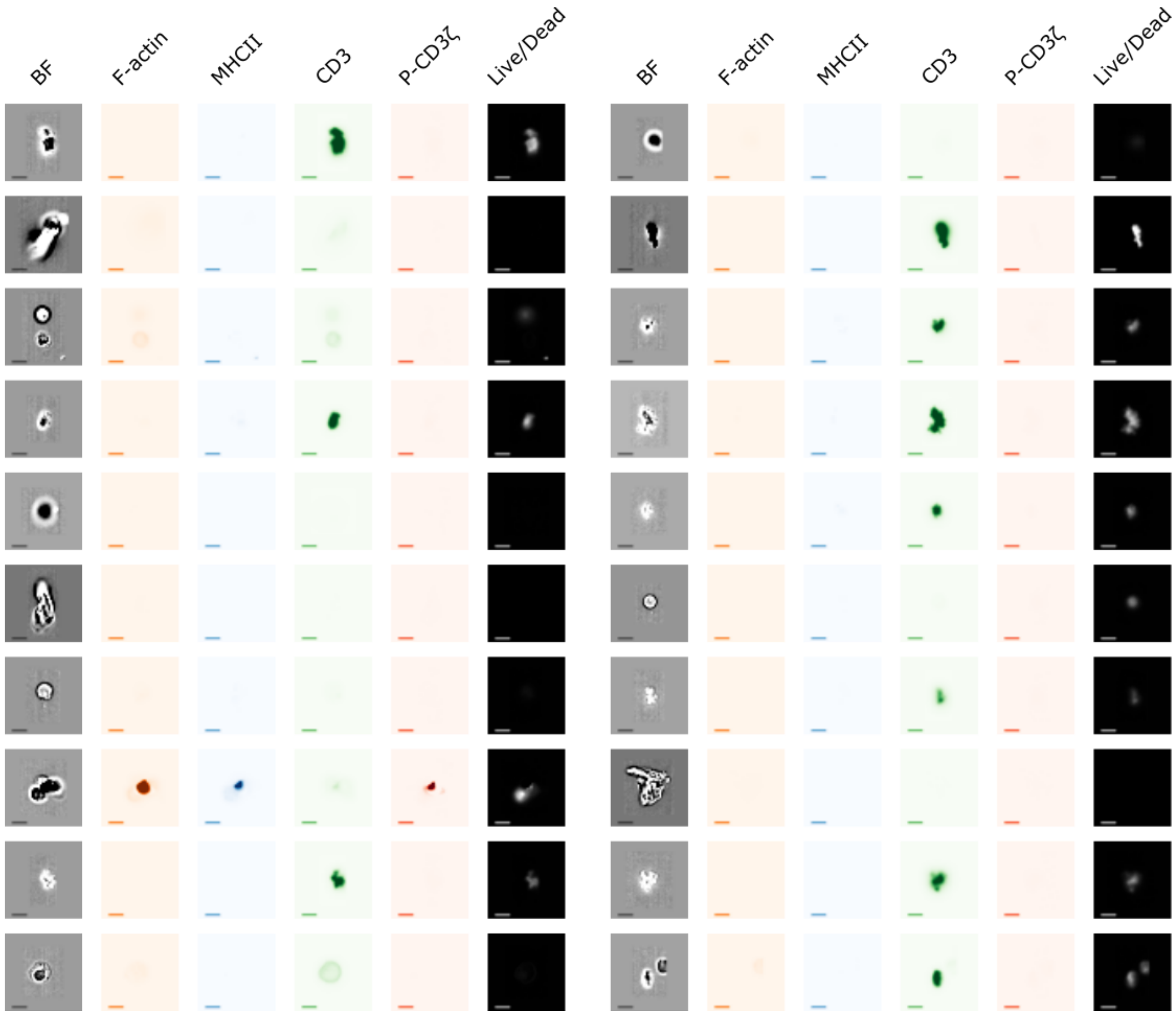

Supplementary Figure 8. Filtered-out images

20 randomly selected examples that were excluded from the analysis based on the data cleaning pipeline described in Methods. The examples show dead cells (with signal in the “Live/Dead” channel), out-of-focus images and images with irregular shapes in the brightfield channel, or missing fluorescent expression (scale bar = 2.4μm)

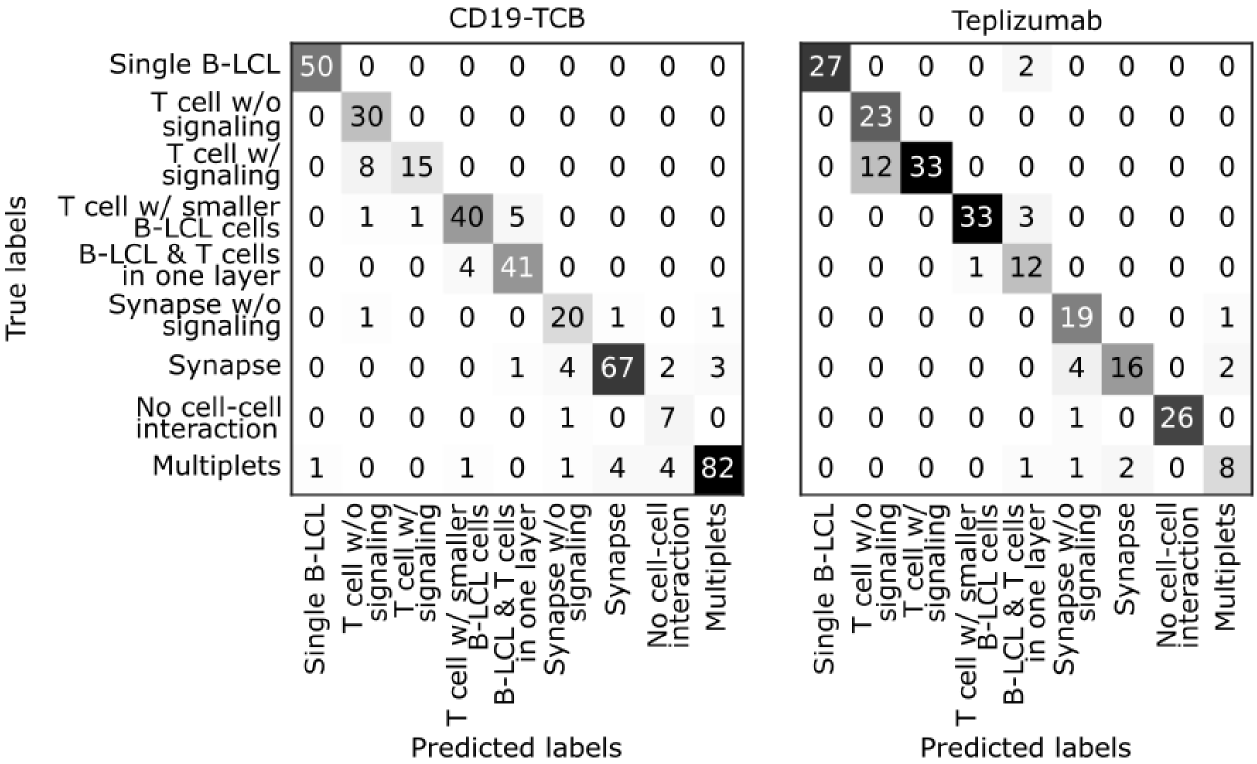

Supplementary Figure 9. Inter-experiment and inter-stimulation generalizability

Confusion matrix for classifications in CD19-TCB and Teplizumab based on 396 and 227 expert-annotated images, respectively. The previously trained model (Fig. 1c) reached a macro F1-score of 0.86 and 0.85, respectively, on both datasets.

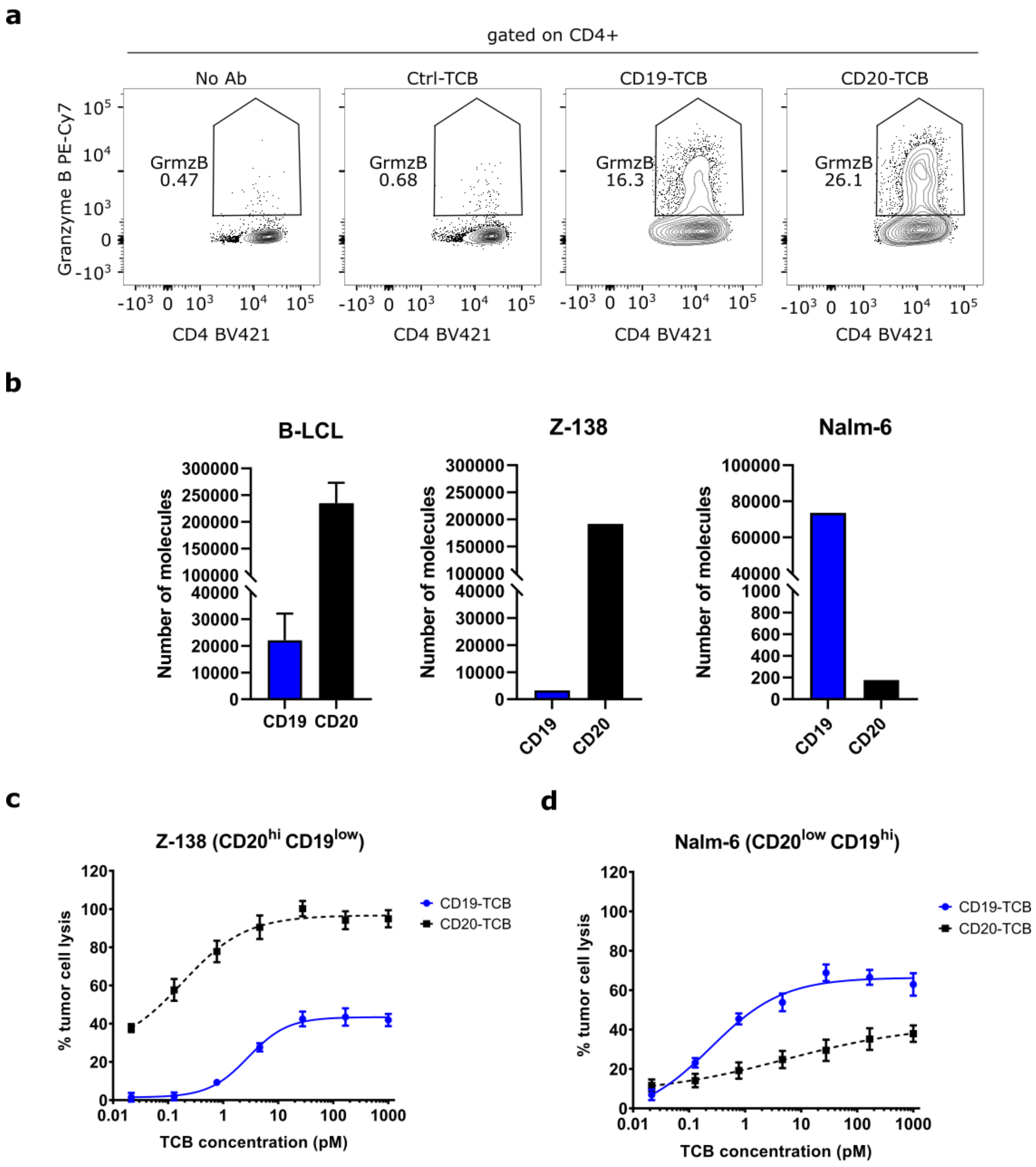

Supplementary Figure 10. Investigation of GrzmB release and tumor-killing induced by CD19- and CD20-TCB.

a FACS plot showing $GrzmB^{+}$ $CD4^{+}$ T cells after treating the cells with Ctrl-TCB, CD19-TCB, or CD20-TCB for 24h or left untreated (no Ab). A representative example of the gating strategy is shown in Supplementary Fig. 11.

b Number of CD19 and CD20 molecules expressed on B-LCL, Nalm-6 and Z-138 cells. The measured values are derived from cell culture and were not measured in parallel to the experiment.

c,d Tumor cell lysis induced by CD19- and CD20-TCB, determined using LDH release after 24 h incubation of B-cell depleted human PBMCs with the tumor targets Nalm-6 or Z-138 and indicated TCB concentrations. One donor is shown.

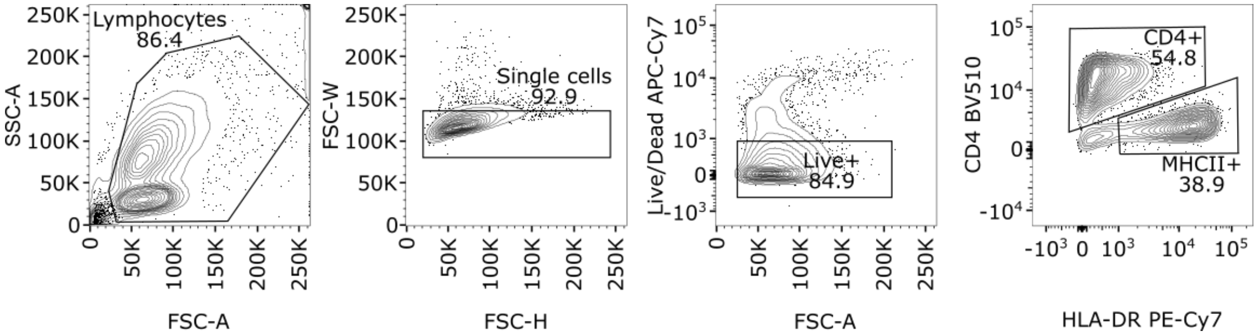

Supplementary Figure 11. Gating strategy of conventional flow cytometry data

Representative example of the gating strategy used for analyzing conventional flow cytometry data as shown in Supplementary Fig. 1d and Supplementary Fig. 10a. Briefly, lymphocytes were selected in the FSC-A and SSC-A gate. In the next step, single cells were selected using FSC-H/FSC-W, and viable cells were identified using the fixable viability dye eF780 (gated on eF780 negative cells). Finally, cells were gated on $CD4^+$ T cells.

feature	feature_type	E3 D5	E4 D2	E4 D8	E4 D7	E4 D9	E3 D1
homogeneity_CD3	texture	0	-1	-1	-1	-1	0
homogeneity_P-CD3zeta	texture	1	1	1	0	1	1
dissimilarity_P-CD3zeta	texture	-1	-1	-1	0	-1	-1
mean_intensity_ratio_CD3_R5_R7	synaptic feature	0	1	1	1	1	0
sum_intensity_ratio_CD3_R5_R7	synaptic feature	0	1	1	1	1	0
mean_intensity_ratio_P-CD3zeta_R5_R7	synaptic feature	0	1	1	1	1	0
sum_intensity_ratio_P-CD3zeta_R5_R7	synaptic feature	0	1	1	1	1	0
sum_intensity_ratio_MHCII_R5_R7	synaptic feature	1	1	1	1	1	0
mean_intensity_ratio_MHCII_R5_R7	synaptic feature	1	1	1	1	1	0
max_intensity_ratio_MHCII_R5_R7	synaptic feature	1	1	1	0	1	1
mask_based_solidity_MHCII	morphology	-1	-1	-1	-1	-1	-1
mask_based_extent_MHCII	morphology	-1	0	-1	-1	-1	0
mask_based_eccentricity_CD3	morphology	1	1	0	1	1	0
mask_based_solidity_CD3	morphology	-1	-1	0	-1	-1	0
mask_based_eccentricity_P-CD3zeta	morphology	1	1	0	1	1	0
mask_based_solidity_P-CD3zeta	morphology	-1	-1	0	-1	-1	0
std_intensity_CD3	intensity statistics	0	0	-1	-1	-1	-1
kurtosis_intensity_P-CD3zeta	intensity statistics	1	1	0	0	1	1
sum_intensity_P-CD3zeta	intensity statistics	1	1	0	1	0	1
mean_intensity_P-CD3zeta	intensity statistics	1	1	1	1	1	1
skew_intensity_P-CD3zeta	intensity statistics	1	1	1	0	1	1
shannon_entropy_P-CD3zeta	intensity statistics	1	1	1	1	1	-1
std_intensity_P-CD3zeta	intensity statistics	1	1	1	1	1	-1
max_intensity_P-CD3zeta	intensity statistics	1	1	1	1	1	1
euclidean_distance_R6_CD3_R7_P-CD3zeta	colocalization	0	0	-1	-1	-1	-1
intensity_correlation_quotient_R5_MHCII_R7_P-CD3zeta	colocalization	0	1	1	1	1	0
intensity_correlation_quotient_R7_P-CD3zeta_R5_MHCII	colocalization	0	1	1	1	1	0

intensity_correlation_quotient_R5_MHCII_R6_CD3	colocalization	0	1	1	1	1	0
euclidean_distance_R7_P-CD3zeta_R6_CD3	colocalization	0	0	-1	-1	-1	-1
correlation_distance_R5_MHCII_R6_CD3	colocalization	-1	-1	-1	-1	-1	0
correlation_distance_R6_CD3_R5_MHCII	colocalization	-1	-1	-1	-1	-1	0
jaccard_distance_MHCII_CD3	colocalization	-1	-1	-1	-1	-1	0
dice_distance_MHCII_CD3	colocalization	-1	-1	-1	-1	-1	0
correlation_distance_R7_P-CD3zeta_R5_MHCII	colocalization	-1	-1	-1	-1	-1	0
correlation_distance_R5_MHCII_R7_P-CD3zeta	colocalization	-1	-1	-1	-1	-1	0
jaccard_distance_MHCII_P-CD3zeta	colocalization	-1	-1	-1	-1	-1	0
dice_distance_MHCII_P-CD3zeta	colocalization	-1	-1	-1	-1	-1	0
correlation_distance_R7_P-CD3zeta_R6_CD3	colocalization	-1	-1	-1	-1	0	-1
correlation_distance_R6_CD3_R7_P-CD3zeta	colocalization	-1	-1	-1	-1	0	-1
correlation_distance_R5_MHCII_R4_F-Actin	colocalization	-1	-1	-1	-1	-1	-1
correlation_distance_R4_F-Actin_R5_MHCII	colocalization	-1	-1	-1	-1	-1	-1
intensity_correlation_quotient_R6_CD3_R5_MHCII	colocalization	0	1	1	1	1	0

Supplementary Table 1. Significant features induced by CD19-TCB
The table represents the features that significantly changed for at least four donors due to stimulation by CD19-TCB. The table represents the list of consistent features from Fig. 3a. 1 represents a significant increase (red in Fig. 3a), -1 represents a significant decrease (blue in Fig. 3a), and 0 represents no significant change (gray in Fig. 3a). The significance is measured by a two-sided Mann-Whitney U test and corrected by the Benjamini-Hochberg procedure. In each column, the number of ‘synapse w/ signaling’ for every donor is: donor 5 experiment 3 (Ctrl-TCB=86, CD19-TCB=660), donor 2 experiment 4 (265, 729), donor 8 experiment 4 (194, 753), donor 7 experiment 4 (96, 822), donor 9 experiment 4 (169, 746) and donor 1 experiment 3 (77, 666).

feature	feature_type	E3 D6	E1 D1	E3 D1	E1 D2	E2 D3	E3 D5	E2 D4
homogeneity_F-Actin	texture	-1	-1	-1	1	-1	-1	1
ASM_F-Actin	texture	-1	-1	-1	1	-1	-1	1
energy_F-Actin	texture	-1	-1	-1	1	-1	-1	1
max_intensity_ratio_F-Actin_R5_R7	synaptic feature	0	-1	-1	1	0	-1	-1
mean_intensity_F-Actin	intensity statistics	-1	-1	-1	1	-1	-1	1
std_intensity_F-Actin	intensity statistics	-1	-1	-1	1	-1	-1	1
min_intensity_F-Actin	intensity statistics	-1	-1	-1	1	-1	-1	1
max_intensity_F-Actin	intensity statistics	-1	-1	-1	1	-1	-1	1
sum_intensity_F-Actin	intensity statistics	-1	-1	-1	1	-1	-1	1
min_intensity_P-CD3zeta	intensity statistics	1	1	1	-1	1	1	0
shannon_entropy_F-Actin	intensity statistics	-1	-1	-1	1	-1	-1	1
manders_overlap_coefficient_R4_F-Actin_R7_P-CD3zeta	colocalization	1	1	1	-1	1	1	-1
manders_overlap_coefficient_R7_P-CD3zeta_R4_F-Actin	colocalization	1	1	1	-1	1	1	-1
euclidean_distance_R4_F-Actin_R7_P-CD3zeta	colocalization	-1	-1	-1	1	-1	-1	1
euclidean_distance_R7_P-CD3zeta_R4_F-Actin	colocalization	-1	-1	-1	1	-1	-1	1
manders_overlap_coefficient_R4_F-Actin_R5_MHCII	colocalization	1	1	1	-1	-1	1	-1
manders_overlap_coefficient_R5_MHCII_R4_F-Actin	colocalization	1	1	1	-1	-1	1	-1

Supplementary Table 2. Significant features induced by Teplizumab

The table represents the features that significantly changed for at least six donors due to stimulation by Teplizumab. The table represents the list of consistent features from Fig. 3h. 1 represents a significant increase (red in Fig. 3h), -1 represents a significant decrease (blue in Fig. 3h), and 0 represents no significant change (gray in Fig. 3h). The significance is measured by a two-sided Mann-Whitney U test and corrected by the Benjamini-Hochberg procedure. In each column, the number of 'synapse w/ signaling' for every donor is: donor 6 experiment 3 (isotype=204, Teplizumab=128), donor 1 experiment 1 (254, 119), donor 1 experiment 3 (328, 222), donor 2 experiment 1 (265, 89), donor 3 experiment 2 (421, 288), donor 5 experiment 3 (326, 227), donor 4 experiment 2 (480, 285)

APC	Antigen-presenting cell
BF	Brightfield
EBV	Epstein-Barr virus
FACS	Fluorescence-activated cell sorting
GrzmB	Granzyme B
HPC	High Performance Computing
IFC	Imaging flow cytometry
LDA	Linear Discriminant Analysis
LR	Logistic Regression
MHC	Major histocompatibility complex
MSE	Mean squared error
PBMCs	Peripheral blood mononuclear cells
RF	Random Forest
RGB	Red Green Blue
SEA	Staphylococcus aureus enterotoxin A
SVM	Support Vector Machine
TCB	T cell bispecific
TCR	T cell receptor
UMAP	Uniform Manifold Approximation and Projection for Dimension Reduction

Supplementary Table 3. Glossary
The complete list of all the abbreviations in the text
