# Supplementary material for: Explainable machine learning for profiling the immunological synapse and functional characterization of therapeutic antibodies
Source: Nat Commun. 2023 Nov 30;14:7888. doi: 10.1038/s41467-023-43429-2 (PMC10689847; doi:10.1038/s41467-023-43429-2)
Supplement: Supplementary file 3 — Reporting Summary [file 41467_2023_43429_MOESM3_ESM.pdf]

nature portfolio

Corresponding author(s): Elke Glasmacher
Carsten Marr
Fabian Schmich

Last updated by author(s): Oct 11, 2023

Reporting Summary

Nature Portfolio wishes to improve the reproducibility of the work that we publish. This form provides structure for consistency and transparency in reporting. For further information on Nature Portfolio policies, see our Editorial Policies and the Editorial Policy Checklist.

Statistics

For all statistical analyses, confirm that the following items are present in the figure legend, table legend, main text, or Methods section.

n/a	Confirmed	
☐	☒	The exact sample size (*n*) for each experimental group/condition, given as a discrete number and unit of measurement
☐	☒	A statement on whether measurements were taken from distinct samples or whether the same sample was measured repeatedly
☐	☒	The statistical test(s) used AND whether they are one- or two-sided<br>*Only common tests should be described solely by name; describe more complex techniques in the Methods section.*
☐	☒	A description of all covariates tested
☐	☒	A description of any assumptions or corrections, such as tests of normality and adjustment for multiple comparisons
☐	☒	A full description of the statistical parameters including central tendency (e.g. means) or other basic estimates (e.g. regression coefficient) AND variation (e.g. standard deviation) or associated estimates of uncertainty (e.g. confidence intervals)
☐	☒	For null hypothesis testing, the test statistic (e.g. *F*, *t*, *r*) with confidence intervals, effect sizes, degrees of freedom and *P* value noted<br>*Give P values as exact values whenever suitable.*
☒	☐	For Bayesian analysis, information on the choice of priors and Markov chain Monte Carlo settings
☒	☐	For hierarchical and complex designs, identification of the appropriate level for tests and full reporting of outcomes
☐	☒	Estimates of effect sizes (e.g. Cohen's *d*, Pearson's *r*), indicating how they were calculated

Our web collection on statistics for biologists contains articles on many of the points above.

Software and code

Policy information about availability of computer code

Data collection	Data was collected by ImageStreamX Mark II Imaging Flow Cytometer (Luminex) and it was exported using the IDEAS 6.2 software from Luminex. The rest of the data pre-processing has be done in python and the detailed description of it is provided in our git repository.
Data analysis	The data analysis is done using python and based on our module, scifAI. This information is also provided in the manuscript. A comprehensive documentation is provided at https://github.com/marrlab/scifAI and https://github.com/marrlab/scifAI-notebooks<br>They were implemented using NumPy (version=1.18.5), Pandas (1.1.5), SciPy (1.8.0), scikit-image (0.19.2), and scikit-learn(1.0.2)

For manuscripts utilizing custom algorithms or software that are central to the research but not yet described in published literature, software must be made available to editors and reviewers. We strongly encourage code deposition in a community repository (e.g. GitHub). See the Nature Portfolio guidelines for submitting code & software for further information.

Data

Policy information about availability of data

All manuscripts must include a data availability statement. This statement should provide the following information, where applicable:
- Accession codes, unique identifiers, or web links for publicly available datasets
- A description of any restrictions on data availability
- For clinical datasets or third party data, please ensure that the statement adheres to our policy

The data set is published and publicly available with Data Dryad at https://doi.org/10.5061/dryad.ht76hdrk7

All the figures data is also provided in https://github.com/marrlab/scifAI-notebooks

Human research participants

Policy information about studies involving human research participants and Sex and Gender in Research.

Reporting on sex and gender	PBMCs were isolated from blood of healthy donors and memory CD4+ T cells were isolated for further characterization. The sex and age of all donors can be found in Supplementary Figure 3a.
Population characteristics	Healthy donors, born between 1955-1995
Recruitment	Healthy donors donate blood on volunteer basis at the internal Medical Service from Roche Diagnostics GmbH at Penzberg, Germany. The donors were volunteers who gave informed consent for experimental research work and were selected based on availability and independent of age and gender by the Medical Service. Material has been anonymised and cannot be tracked back to the original donor.
Ethics oversight	Approval of the ethics committee of the Bayerische Landesärztekammer.

Note that full information on the approval of the study protocol must also be provided in the manuscript.

Field-specific reporting

Please select the one below that is the best fit for your research. If you are not sure, read the appropriate sections before making your selection.

[x] Life sciences [ ] Behavioural & social sciences [ ] Ecological, evolutionary & environmental sciences

For a reference copy of the document with all sections, see nature.com/documents/nr-reporting-summary-flat.pdf

Life sciences study design

All studies must disclose on these points even when the disclosure is negative.

Sample size	The dataset includes 9 donors based on four experiments, three antibodies. The donors were selected randomly based on their availability. Considering that the donors were selected to only show the use-case of our pipeline, no sample size calculation was done.
Data exclusions	No data exclusion was done.
Replication	All replicates, number of donors per experiment, their age and sex are described in the Supplementary Figure 3a-d. All the attempts for replication were successful. No other replicate was done.
Randomization	The donor selection for each separate experiment was random and performed based on the availability of donors and independent on age and gender. Considering that the donors were selected to only show the use-case of our pipeline, no sample size calculation was done.
Blinding	Considering that the donors were selected to only show the use-case of our pipeline, no blinding was needed for our study.

Reporting for specific materials, systems and methods

We require information from authors about some types of materials, experimental systems and methods used in many studies. Here, indicate whether each material, system or method listed is relevant to your study. If you are not sure if a list item applies to your research, read the appropriate section before selecting a response.

Materials & experimental systems

n/a	Involved in the study	
[ ]	[x]	Antibodies
[ ]	[x]	Eukaryotic cell lines
[x]	[ ]	Palaeontology and archaeology
[x]	[ ]	Animals and other organisms
[x]	[ ]	Clinical data
[x]	[ ]	Dual use research of concern

Methods

n/a	Involved in the study	
[x]	[ ]	ChIP-seq
[ ]	[x]	Flow cytometry
[x]	[ ]	MRI-based neuroimaging

Antibodies

Antibodies used

CD3-BV421 clone UCHT1, Biolegend; cat # 300434; Lot: B359906; 1:20
CD4-BV510 clone RPA-T4, Biolegend: cat # 300546; Lot:not available; 1:100
CD4-BV421 clone RPA-T4, BD; cat # 562424; Lot: 0084457; 1:50
CD69-PE clone FN50, Biolegend; cat # 310906; Lot: B258744; 1:200
HLA-DR-PE-Cy7 clone L243, Biolegend; cat # 307616; Lot: B281898; 1:200
Phalloidin AF594 ThermoFisher; cat # A12381; Lot 2256805; 1:600
P-CD3ζ Y142-AF647 clone K25-407.69, BD cat # 558489; Lot 8262873; 1:20
Granzyme B-PE-Cy7 clone QA16A02, Biolegend; cat # 372214; Lot: B368220 ; 1:50
TNF-α clone MAb11, BD; cat # 554514; Lot 2312292; 1:50
CD80-APC clone 2D10, Biolegend cat # 305220; Lot: B229474; 1:200
CD86-PE clone IT2.2, Biolegend cat # 305406; Lot: B210795; 1:200
Teplizumab; in-house; 10 µg/mL
CD19-TCB; in-house; 1 µg/mL
CD20-TCB; in-house; 1 µg/mL

Validation

We provided a link for the relevant information and data sheet for each antibody that was used for flow cytometry. The data sheet includes the validation of all primary antibodies for the species and application:
CD3-BV421 clone UCHT1: https://www.biolegend.com/en-us/products/brilliant-violet-421-anti-human-cd3-antibody-7153?GroupID=BLG5900
CD4-BV510 clone RPA-T4: https://www.biolegend.com/en-us/products/brilliant-violet-510-anti-human-cd4-antibody-9598
CD4-BV421 clone RPA-T4: https://www.bdbiosciences.com/en-us/products/reagents/flow-cytometry-reagents/research-reagents/single-color-antibodies-ruo/bv421-mouse-anti-human-cd4.562424
CD69-PE clone FN50: https://www.biolegend.com/en-us/products/pe-anti-human-cd69-antibody-1672?GroupID=BLG10251
HLA-DR-PE-Cy7 clone L243: https://www.biolegend.com/en-us/products/pe-cyanine7-anti-human-hla-dr-antibody-2862?GroupID=BLG5955
Phalloidin AF594: https://www.thermofisher.com/order/catalog/product/de/en/A12381
P-CD3ζ Y142-AF647 clone K25-407.69: https://www.bdbiosciences.com/en-de/products/reagents/flow-cytometry-reagents/research-reagents/single-color-antibodies-ruo/alexa-fluor-647-mouse-anti-cd247-py142.558489
Granzyme B-PE-Cy7 clone QA16A02: https://www.biolegend.com/en-us/products/pe-cyanine7-anti-humanmouse-granzyme-b-recombinant-antibody-15582?GroupID=GROUP28
TNF-α clone MAb11: https://www.bdbiosciences.com/en-de/products/reagents/flow-cytometry-reagents/research-reagents/single-color-antibodies-ruo/apc-mouse-anti-human-tnf.554514
CD80-APC clone 2D10: https://www.biolegend.com/fr-ch/soluble-mhc/apc-anti-human-cd80-antibody-6530?GroupID=BLG1908
CD86-PE clone IT2.2: https://www.biolegend.com/en-us/products/pe-anti-human-cd86-antibody-778

Teplizumab, CD19-TCB and CD20-TCB were produced in-house and their identity was confirmed by mass spectrometry. A detailed description of the antibodies can be found in the manuscript in the results part. CD19-TCB and CD20-TCB were validated in previous studies: Leclercq, G. et al. JAK and mTOR inhibitors prevent cytokine release while retaining T cell bispecific antibody in vivo efficacy. J Immunother Cancer 10, e003766 (2022) & Marina, B. et al. Bacac et al., Clinc Cancer Res 2018, CD20-TCB with Obinutzumab pretreatment as next-generation treatment of hematologic malignancies .pdf. Clin Cancer Res 24, 4785 (2018). Additionally, CD20-TCB is clinically validated (Birtas Atesoglu, E, Gulbas, Z, Uzay, A, et al. Glofitamab in relapsed/refractory diffuse large B-cell lymphoma: real-world data. Hematol Oncol. 2023; 1-11. https://doi.org/10.1002/hon.3174) and a health authorities approved drug: https://www.fda.gov/drugs/drug-approvals-and-databases/fda-grants-accelerated-approval-glofitamab-gxbm-selected-relapsed-or-refractory-large-b-cell & https://www.ema.europa.eu/en/medicines/human/EPAR/columvi. CD19-TCB is currently under investigation in a clinical phase I study NCT05835986.

Eukaryotic cell lines

Policy information about cell lines and Sex and Gender in Research

Cell line source(s)

EBV-transformed B-lymphoblastoid cell line (B-LCL) from donor 333 from Astarte Biologics (# 1038-3161JN16). The sex of donor 333 is female and the age is 25 years. Z138 (MCL, gift from University of Leicester but can be also obtained from ATCC) and Nalm-6 (ALL, DSMZ ACC 128) are tumor cell lines.

Authentication

The cell lines were authenticated by morphology on a regular basis by our internal cell data bank from Roche Pharmaceutical Research and Early Development (pRED), Roche Innovation Center Munich. In addition, Z-138 cell line was authenticated by STR profiling by Microsynth and Nalm-6 was purchased from DSMZ (German Collection of Microorganisms and Cell Cultures GmbH) who runs STR profiling on their cell lines.

Mycoplasma contamination

All cell lines were tested negative for mycoplasma contamination. The cell lines are regularly analyzed by our internal cell data bank from Roche Pharmaceutical Research and Early Development (pRED), Roche Innovation Center Munich.

Commonly misidentified lines
(See ICLAC register)

No misidentified cell lines were used.

Flow Cytometry

Plots

Confirm that:

- [x] The axis labels state the marker and fluorochrome used (e.g. CD4-FITC).
- [x] The axis scales are clearly visible. Include numbers along axes only for bottom left plot of group (a 'group' is an analysis of identical markers).
- [x] All plots are contour plots with outliers or pseudocolor plots.
- [x] A numerical value for number of cells or percentage (with statistics) is provided.

Methodology

Sample preparation	see manuscript, Methods
Instrument	ImageStreamX Mark II Imaging Flow Cytometer (Luminex); FACS Celesta (BD Biosciences)
Software	IDEAS 6.2 and FlowJo 10.8.1
Cell population abundance	Purity check of isolated memory CD4+ T cells from PBMCs was performed for each experiment. The purity was between 88-94%.
Gating strategy	The gating strategy to identify immune synapses is shown in the Supplementary Fig. 1a. A representative example of the gating strategy used for analyzing conventional flow cytometry data is shown in Supplementary Fig. 9a and is described in the Methods part.

- [x] Tick this box to confirm that a figure exemplifying the gating strategy is provided in the Supplementary Information.
